# Fabrication of metal complex phthalocyanine and porphyrin nanoparticle aqueous colloids by pulsed laser fragmentation in liquid and their potential application to a photosensitizer for photodynamic therapy

**DOI:** 10.3762/bjnano.16.80

**Published:** 2025-07-11

**Authors:** Taisei Himeda, Risako Kunitomi, Ryosuke Nabeya, Tamotsu Zako, Tsuyoshi Asahi

**Affiliations:** 1 Graduate School of Science and Engineering, Ehime University, 3 Bunkyo-cho, Matsuyama, Ehime 790-8577, Japan

**Keywords:** aqueous colloid, photosensitizer, phthalocyanine, pulsed laser fragmentation in liquids, reactive oxygen species generation

## Abstract

We prepared stable nanoparticle dispersions of metal complex phthalocyanines (MPcs; M = AlCl, Fe, Co, Zn) and Pt complex octaethylporphyrin (PtOEP) by nanosecond laser fragmentation of the corresponding microcrystalline powders in an aqueous solution of the amphiphilic polymer Pluronic^®^ F-127. All nanoparticles dispersed stably in phosphate-buffered saline and cell culture media without any precipitation for longer than one week. The aqueous F-127 solution at 0.1 wt % concentration, which is about one tenth of the critical micelle concentration, was enough to fabricate nanoparticles with excellent dispersion stability and high production efficiency. We examined the photosensitized generation of reactive oxygen species by AlClPc, ZnPc, and PtOEP nanoparticles and the photocytotoxicity for PC12 and HeLa cells, and demonstrated that the nanoparticles can be used as photosensitizers for photodynamic therapy.

## Introduction

Porphyrins and phthalocyanines (Pcs), exhibiting intense absorption in the visible to near-infrared (NIR) regions, are well studied as photosensitizers in photodynamic therapy (PDT) [1]. Especially, Pcs absorb strongly light in the biological optical window (wavelengths from 650 to 1000 nm) and have recently attracted attention for applications in biomedical research such as photoacoustic imaging of tissues and PDT of tumors [2–3]. Porphyrins and Pcs are hydrophobic hydrocarbons that are insoluble in water. Hence, polymer composite nanoparticles and nanomicelle encapsulation have been used to disperse them in water [2–7]. For example, AlClPc has been loaded into nanoemulsions using castor oil and Cremophor ELP^®^ [5]. ZnPc was dispersed in unilamellar liposomes by a solvent exchange method [7–8], and its photocytotoxicity against cancer cells was reported. However, conventional methods of producing nanoparticle colloids require organic solvents and excessive amounts of organic adjuvants, which may have other implications for research in pharmacological, photochemical, and medical applications, and also may interfere with the activity of the target substance in general. An alternative and promising method for dispersing hydrophobic organic compounds as colloids is pulsed laser fragmentation in liquids (PLFL) [9–10]. This relatively new fabrication method has advantages because a microcrystalline sample powder suspended in a poor solvent is fragmented into nanoparticles by intense pulsed laser irradiation, and the sample suspension is directly converted in to a colloidal dispersion without any chemical additives in one step. It has been demonstrated that several hydrophobic dyes such as metal complex Pcs (MPcs) [11–17], perylene [18], perylene diimides [19–20], and quinacridones [9,21] can be successfully dispersed in water without the use of organic solvents. Moreover, PLFL has been applied recently to active pharmaceutical ingredients such as vitamin C, capsaicin [22], megestrol acetate [23], paclitaxel [24], naproxen and fenofibrate [25], curcumin [26], and cinnamon [27].

**Figure 1:**
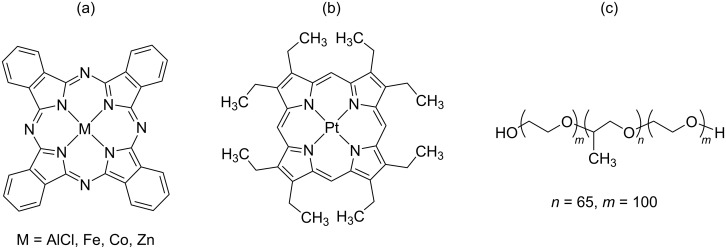
Molecular structures of (a) MPcs, (b) PtOEP, and (c) Pluronic F-127.

This work aims to fabricate aqueous colloids of MPc (M = AlCl, Fe, Co, Zn) and Pt complex octaethylporphyrin (PtOEP) (Figure 1) nanoparticles with a high dispersion stability and to demonstrate the potential application as photosensitizers for PDT. We have already reported the nanoparticle fabrication of some MPcs by PLAL using deionized water [13–15]. The nanoparticles dispersed well in pure water, but precipitated in a buffer solution and a cell culture medium after one day. In this study, therefore, an amphiphilic polymer (Pluronic^®^ F-127, Figure 1) [28] was used as a dispersant to ensure high dispersion stability in various aqueous environments in anticipation of future bio-applications. F-127 is a less toxic and non-ionic copolymer containing hydrophilic polyethylene oxide (PEO) and hydrophobic polypropylene oxide (PPO) arranged in a triblock structure. Here, the stability in phosphate-buffered saline (PBS, pH 7.2), which is used widely in pharmacology and biomedical experiments, was examined. We evaluated the phototoxicity of the fabricated nanoparticle colloids in vitro against PC12 cells (a cell line derived from a pheochromocytoma of the rat adrenal medulla) and HeLa cells, and investigated the photosensitized generation of highly reactive oxygen species (ROS) by the nanoparticles.

## Results and Discussion

**Figure 2:**
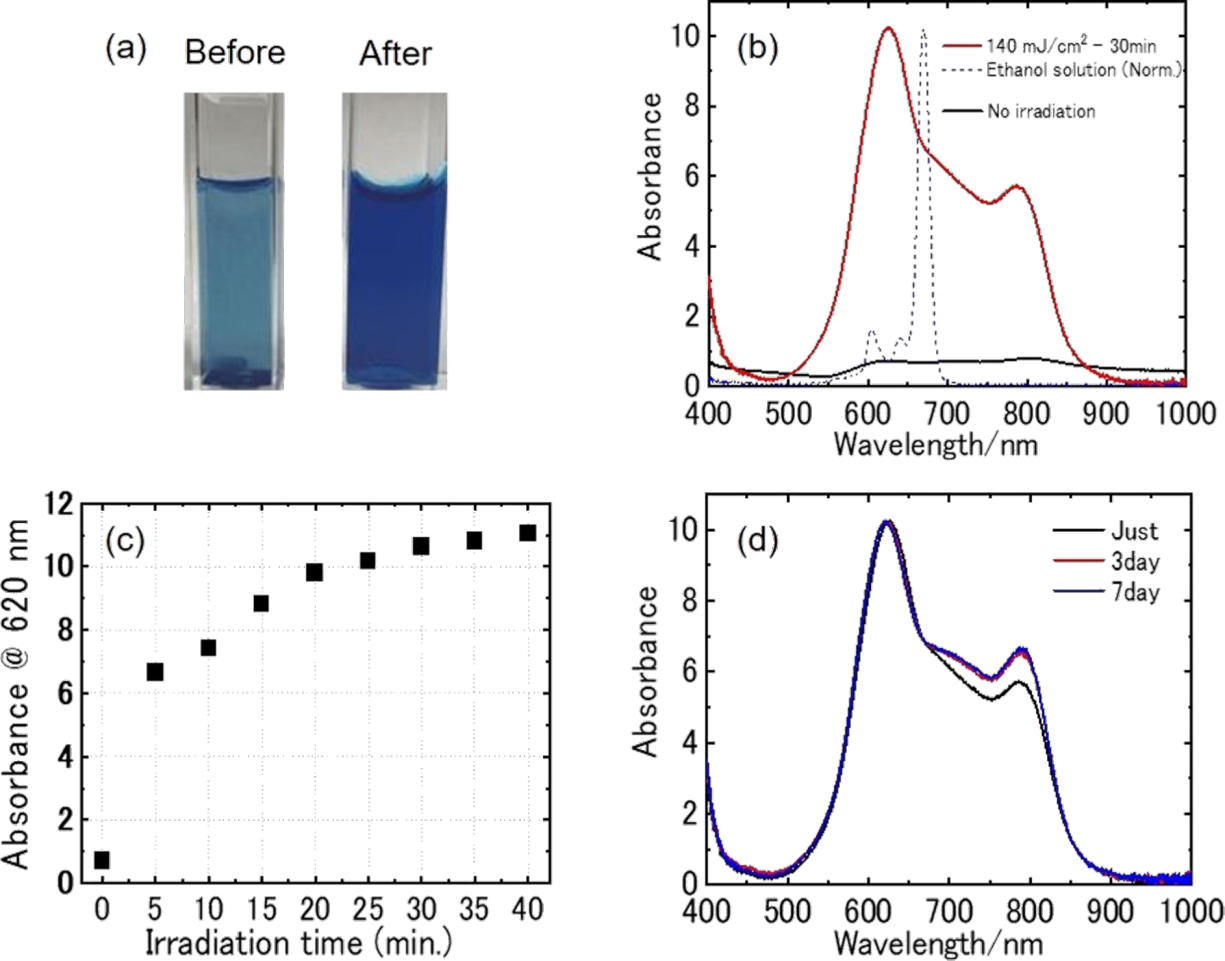
(a) Photographs of the mixture of AlClPc (0.020 wt %) and F-127 (0.1 wt %) aqueous solution before (left) and after (right) nanosecond pulsed laser irradiation (532 nm wavelength, 6 ns full-width at half-maximum, 10 Hz repetition rate) at 140 mJ·cm^−2^ for 30 min. (b) Absorption spectra of the mixture before (black line) and after (red line) laser irradiation. The dashed line shows the absorption spectrum of an AlClPc ethanol solution. (c) Laser irradiation time dependence of absorbance at the wavelength of 620 nm. (d) Absorption spectra of the prepared nanoparticle colloids 10 min (black line), 3 days (red line), and 7 days (blue line) after laser irradiation. Absorption spectra were measured in a 2 mm optical path length cuvette and the absorbance values are shown in terms of 1 cm optical path length.

As a representative example, the results for AlClPc produced by PLFL in a 0.1 wt % concentration of F-127 solution is described in detail in Figure 2. This concentration is about one tenth of the critical micelle concentration (CMC = 0.7 wt % at 25 °C) [29]. The AlClPc/F-127 aqueous suspension (0.02 wt %/0.1 wt %) was sonicated for 20 min. Then, 2 mL of the suspension was placed in a plastic cuvette (1 × 1 × 4 cm^3^) and irradiated with nanosecond laser pulses (532 nm wavelength, 6 ns full-width at half-maximum, 10 Hz repetition rate, fluence = 140 mJ·cm^−2^ per pulse) under stirring with a magnetic stirrer. The nanoparticle generation was examined by measuring the absorption spectrum with a USB spectrometer, and the sample was irradiated with laser pulses until the absorption spectrum stopped changing.

The sample was muddy blue, and most of the AlClPc precipitated to the bottom of the cuvette before laser irradiation. After irradiation with nanosecond laser pulses for 40 min (Figure 2a), the sample turned to deep clear blue. A broad absorption with peaks at 620 and 800 nm appeared and increased with laser irradiation time; the absorbance stopped changing after around 30 min irradiation as shown in Figure 2b. The two peaks that appeared at short and long wavelengths relative to the isolated molecular absorption peak (680 nm) are characteristic of the spectral shape of MPc solids, in which planar molecules stack in slipped arrangements in one dimension. It is well known that the peak wavelengths and the relative absorption intensity of the peaks depends on the crystalline structure, that is, the relative orientation between neighboring molecules. A sharp absorption peak at 680 nm of isolated molecules was not observed, confirming that AlClPc dispersed as nanoparticles in water. The slight change in spectral shape over time (Figure 2d) is probably due to the change in molecular packing within the nanoparticles. The hydrodynamic diameter of the colloidal dispersion was examined by dynamic light scattering (DLS) measurements, and the *Z*-average value was 96 nm (see Figure S2, Supporting Information File 1). The fabricated nanoparticles remained stably dispersed over a period of one month in solution.

**Figure 3:**
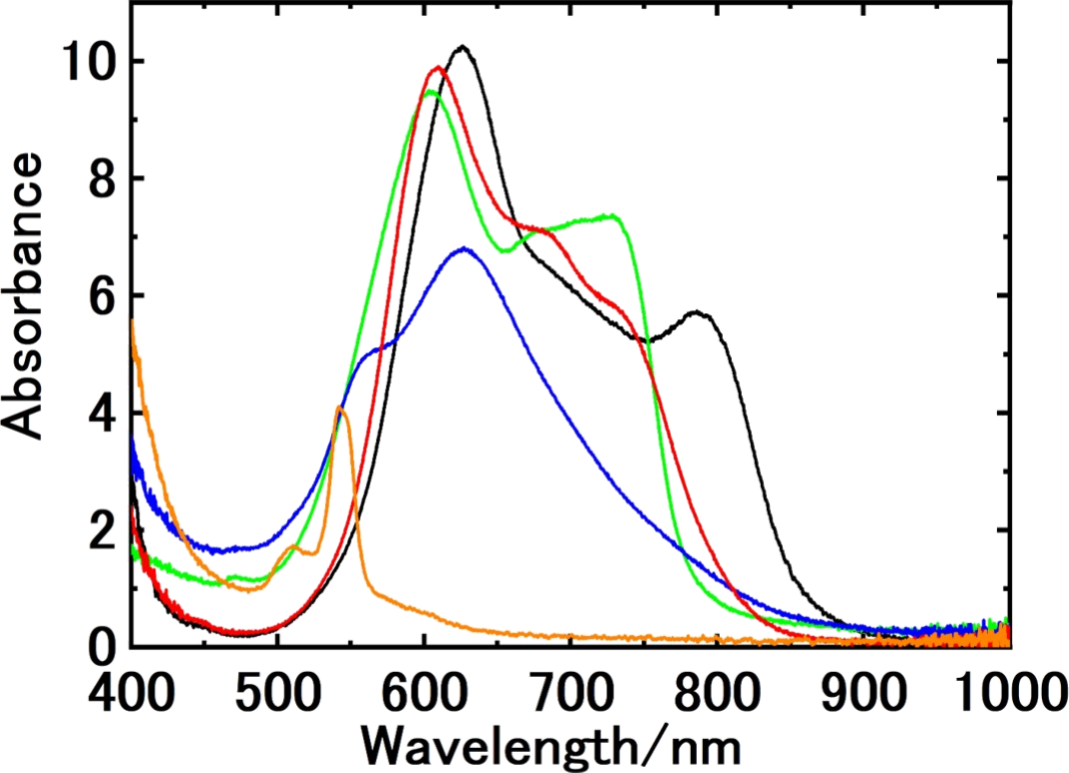
Absorption spectra of the nanoparticle colloids of AlClPc (black line), CoPc (green line), ZnPc (red line), FePc (blue line), and PtOEP (orange line) fabricated by nanosecond pulsed laser irradiation of their microcrystalline powder in F-127 (0.1 wt %) solution at 140 mJ·cm^−2^. Dye concentrations were 0.020 wt % for each sample.

Similar results were obtained for other MPcs and PtOEP prepared in 0.1 wt % F-127 aqueous solution (Figure S1, Supporting Information File 1). The absorption spectra of the nanoparticle colloids are shown in Figure 3, and some characteristics of the colloids are summarized in Table 1. The nanoparticles having strong absorption in the NIR region (700 to 900 nm) were successfully prepared by PLFL, and the colloids remained stably dispersed over a period of at least one week. Particle size and size distribution determined by DLS measurements depended on the molecules. The compound-dependent diameters were estimated to be 40 to 80 nm from the number-weighted size distributions by DLS measurements (see Figure S2, Supporting Information File 1). The *Z*-average values suggest that AlClPc and PtOEP have a narrower size distribution than FePc, CoPc, and ZnPc.

**Table 1:** Some characteristics of the prepared nanoparticles.

	absorption peak wavelength (nm)	absorption coefficient^a^ (× 10^4^ M^−1^·cm^−1^)	particle size (nm)	

			*Z*-average	mean diameter^b^

AlClPc	620	2.9	96	53
CoPc	605	2.7	212	84
FePc	625	1.9	197	63
ZnPc	605	2.8	160	63
PtOEP	550	1.5	90	35

^a^The values were estimated assuming that all raw materials were converted to nanoparticles. ^b^The values were estimated from the number-weighted size distribution by DLS measurements.

In general, molecular degradation may occur during nanoparticle fabrication via PLAL. To investigate molecular degradation, the prepared nanoparticles were dissolved in organic solvents and their visible to NIR absorption spectra were measured. The spectra agreed well with those of the raw material compounds, confirming that at least no organic compounds with absorption in the visible and NIR regions were formed as degradation products. Furthermore, since phthalocyanines and porphyrins are molecules with excellent light and heat resistance, molecular degradation was considered negligible in this study.

**Figure 4:**
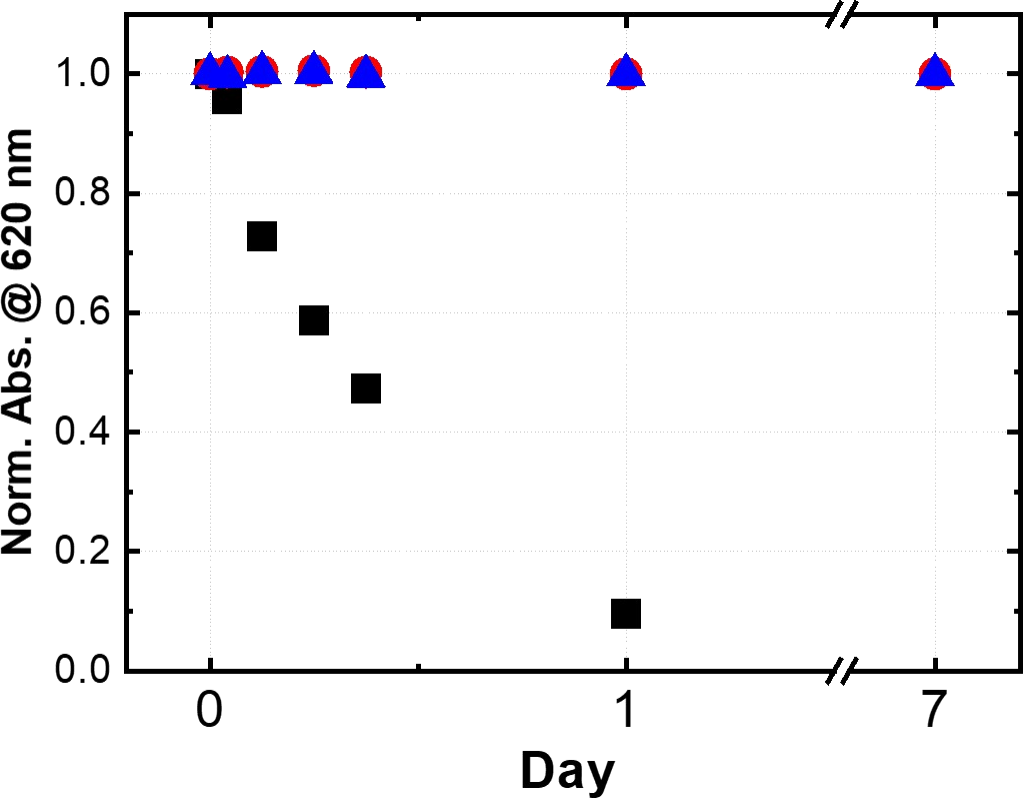
Dispersion stability of the AlClPc nanoparticles fabricated in 0.1 wt % F-127 solution (blue triangles), in pure water without F-127 (black squares), and the pure colloids with F-127 added (red circles) evaluated by absorption measurements. A decrease in absorbance at 620 nm means precipitation of the nanoparticles.

To evaluate the dispersion stability in PBS, 0.2 mL of the prepared colloids was put in 1.8 mL of PBS, and then the absorption spectra were measured after one week. In the case of AlClPc, nanoparticle colloids were efficiently formed, even in pure water suspensions, and the absorption spectra of the prepared nanoparticle colloids were almost the same with and without F-127 (Figure S4, Supporting Information File 1). We compared the stability of the AlClPc colloids prepared in 0.1 wt % F-127 solution with those of the colloids prepared in pure water without F-127 and the pure colloid with F-127 added. As shown in Figure 4, the pure nanoparticles precipitated after one day in PBS, while the nanoparticles with F-127 remained stably dispersed over one week. A similar dispersion stability was observed for cell culture media such as MEM and RPMI1640. Nanoparticles of other MPcs and PtOEP prepared in 0.1 wt % F-127 aqueous solution remained stably dispersed in PBS, as was the case for AlClPc (Figure S4, Supporting Information File 1). The results indicate that the nanoparticles generated by pulsed laser irradiation will be coated tightly with F-127 molecules through hydrophobic interactions between the extremely hydrophobic MPc and the hydrophobic PPO block of F-127, leading to high dispersion stability not only water but also in PBS.

**Figure 5:**
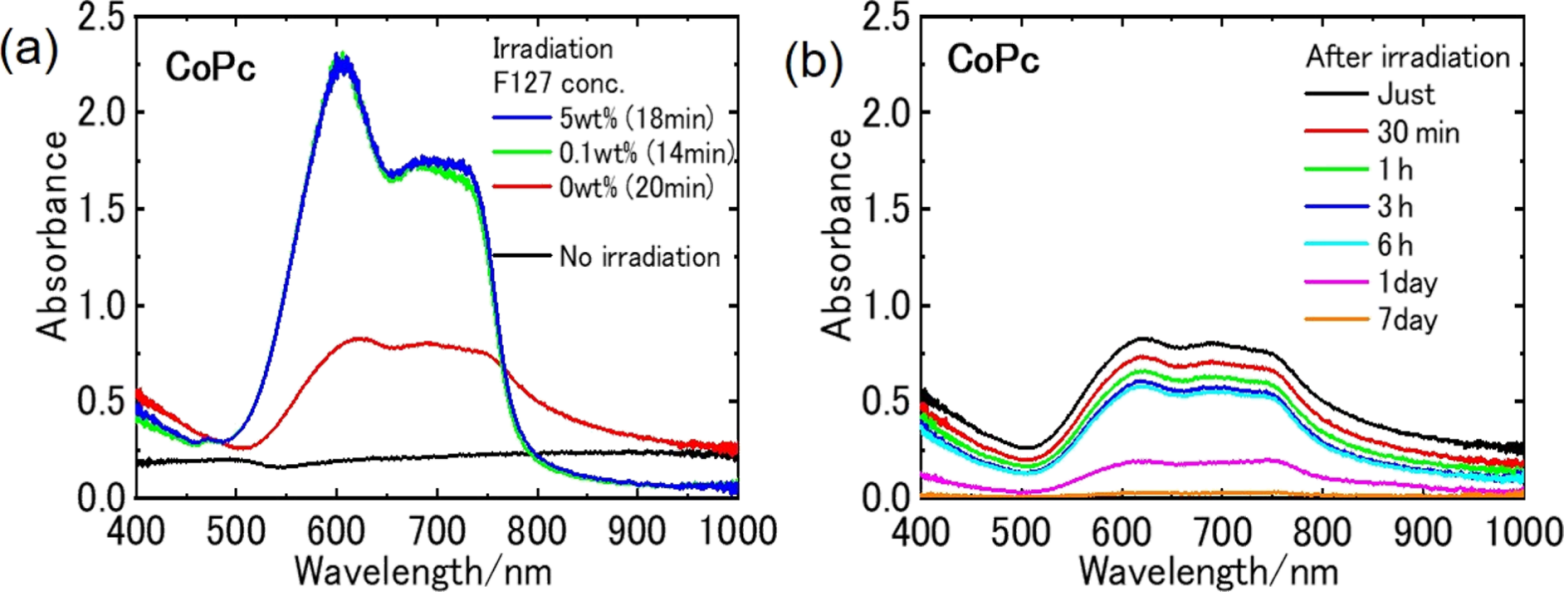
(a) Absorption spectra of the CoPc colloid (0.005 wt %) fabricated in F-127 solution of different concentrations (0, 0.1, and 5 wt %) with a nanosecond pulsed laser at 140 mJ·cm^−2^. (b) Absorption spectra of the pure CoPc colloid without F-127 while standing over the period of one week after preparation.

**Figure 6:**
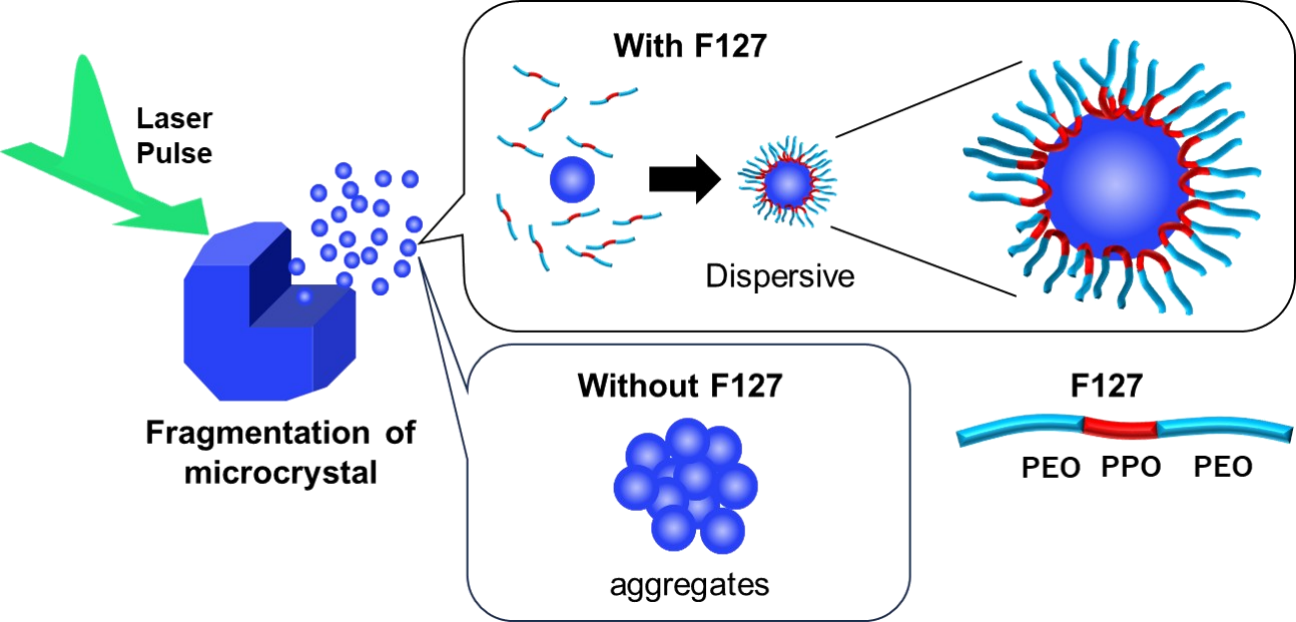
Schematic illustration of dispersible nanoparticle formation by PLAL of microcrystalline powders in F-127 solution.

In cases of other MPcs (M = Co, Fe, Zn), the addition of F-127 significantly influenced the efficiency of nanoparticle generation as well as their dispersion stability (Figure S4, Supporting Information File 1). Absorption spectra of the colloids prepared by irradiating the suspended sample powder (0.005 wt %) in F-127 solutions of different concentrations (0, 0.1, and 5 wt %) with nanosecond pulsed laser at 140 mJ·cm^−2^ were measured, and the results for CoPc are shown in Figure 5 as an example. The absorbance of the sample prepared in pure water was low even after laser irradiation for 20 min and did not increase after further irradiation. The spectra showed a tail at longer wavelengths over 800 nm, and the broad absorption decreased significantly after one day. This suggests that small nanoparticles generated by laser fragmentation are extremely unstable and aggregate rapidly to form larger nanoparticles, which precipitated after one day. In F-127 solutions, in contrast, F-127 molecules coated the surface of the nanoparticles immediately after generation, and small stably dispersed nanoparticles were obtained with a high fabrication efficiency. As the absorption spectra of the colloids prepared in 0.1 and 5 wt % F-127 solutions were the same, a concentration of 0.1 wt %, which is about one tenth that of the CMC of F-127, is enough to prepare stable nanoparticle colloids of MPcs and PtOEP with high efficiency. The role of F-127 in the formation of small nanoparticles having high dispersion stability is schematically illustrated in Figure 6.

**Figure 7:**
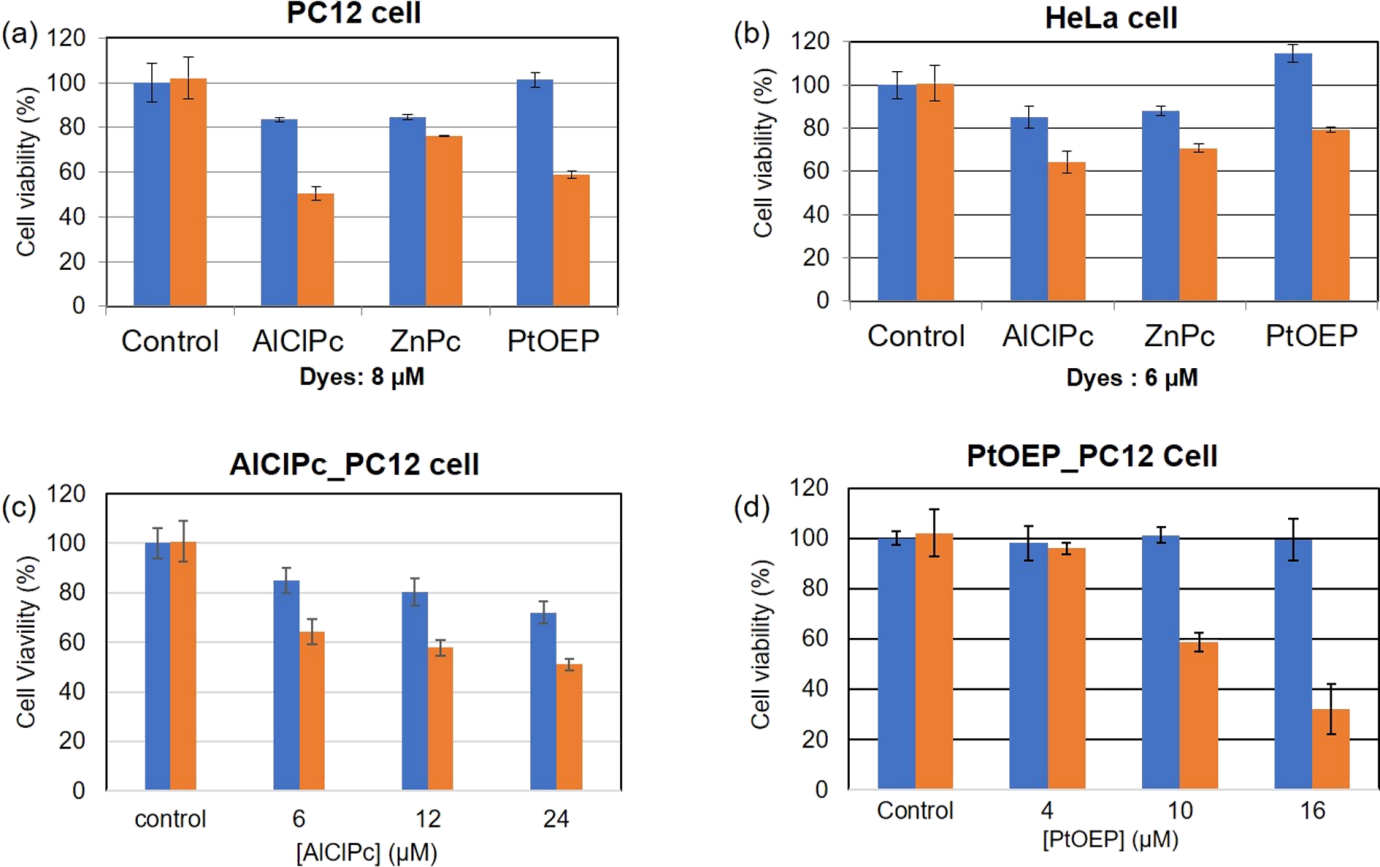
The photocytotoxicity of AlClPc, ZnPc, and PtOEP nanoparticles for PC12 and HeLa cells. Cell viability with (red bar) and without (blue bar) light irradiation (500–800 nm wavelength, 35 mW·cm^−2^ for 10 min) after incubation with the nanoparticles for 24 h was evaluated with MTT assays. (a) PC12 cells: dye concentration of 8 μM. (b) HeLa cells: dye concentration 8 μM. (c, d) Concentration dependence of AlClPc and PtOEP nanoparticles for PC12 cells.

The cellular phototoxicity of the AlClPc, ZnPc, and PtOEP nanoparticles prepared in 0.1 wt % F-127 aqueous solution was examined by employing MTT assays with PC12 and HeLa cells. Nanoparticle colloids diluted with PBS to various concentrations were added to the cell culture medium and incubated for 24 h; then, the cell viability was examined with and without light irradiation by using an MTT cell proliferation kit as described earlier [30–31]. A halogen lamp light with a wide spectral range of 500–800 nm using optical filters was used as light source for 10 min at an output power of 35 mW·cm^−2^ (Figure S6 and Figure S7, Supporting Information File 1). For AlClPc and PtOEP nanoparticles, a significant reduction in the cell viability of PC12 and HeLa cells was observed upon light irradiation, while slight toxicity was observed for MPcs even in the dark (Figure 7). Both AlClPc and PtOEP demonstrated dose-dependent photocytotoxicity for PC12 cells.

**Figure 8:**
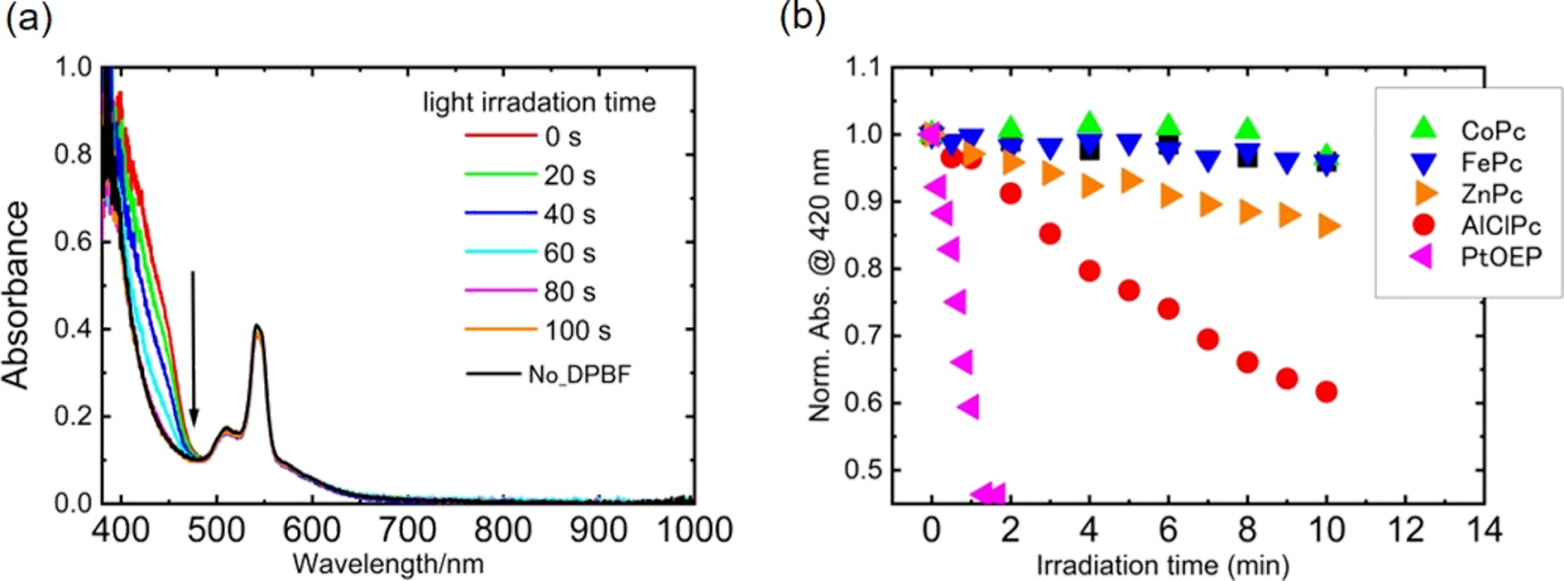
Absorption spectra of the mixture (1 mL) of PtOEP nanoparticles (dye concentration ca. 30 μM) and DPBF (30 μM) before (red line) and after (blue line) light irradiation (500–800 nm wavelength) at 40 mW·cm^−2^ for 100 s. (b) Decay profiles of DPBF absorption at 420 nm for AlClPc (red), ZnPc (orange), CoPc (green), FePc (blue), and PtOEP (pink). The value of absorbance is normalized to that before light irradiation for each sample.

To investigate the photocytotoxicity properties of the nanoparticles, the photosensitized ROS generation was examined by using 1,3-diphenylisobenzofuran (DBPF) as chemical quencher. DPBF is a substance that reacts efficiently with ROS such as singlet oxygen, hydroxyl radicals, and superoxide anions [32–34]. The generation of ROS was monitored by absorption spectroscopy of the photolysis of DPBF. For this analysis, a mixture (1 mL) of the MPc or PtOEP nanoparticles (dyes concentration ca. 30 μM) and DPBF (30 μM) was irradiated with the same light source (500–800 nm) as in the phototoxicity experiment at 40 mW·cm^−2^; the results obtained for PtOEP are shown in Figure 8a as an example. Light irradiation caused a decrease in absorbance around 420 nm, which corresponds to the absorption band of DPBF, indicating the generation of ROS. In contrast, the absorption of PtOEP nanoparticles did not change, indicating that these nanoparticles have high photostability. We compared the ROS generation with a conventional water-soluble photosensitizer, Zn(II) *meso*-tetra(4-sulfonatophenyl)porphine tetrasodium salt (ZnTPPS), and demonstrated that the PtOEP nanoparticles acted as photosensitizers with greater efficiency than ZnTPPS (Figure S8, Supporting Information File 1).

The decays of DPBF absorption for MPcs and PtOEP nanoparticles are summarized in Figure 8b. The ROS generation was confirmed for AlClPc and ZnPc, while it was not confirmed for CoPc and FePc. From the decay rate of DPBF, the ROS generation efficiency was evaluated and it was in the order of PtOEP > AlClPc > ZnPc. This result strongly supports that cellular phototoxicity is due to photosensitized ROS generation by the nanoparticles. Therefore, it is concluded that the MPc and PtOEP nanoparticles fabricated by PLAL could be used as a new potential photosensitizer for PDT. The efficiency for PDT in acting as a sensitizer cannot be properly assessed at this stage of the study. This is because the evaluation of cellular phototoxicity requires consideration of cellular uptake and localization, and further studies must be conducted.

## Conclusion

We successfully fabricated several MPc (M = AlCl, Fe, Co, Zn) and PtOEP nanoparticles by nanosecond laser fragmentation in Pluronic F-127 (0.1 wt %) aqueous solution. The MPc nanoparticles exhibited a strong absorption band in NIR. The prepared nanoparticles with a hydrodynamic diameter of about 60 nm dispersed stably in PBS and cell culture media without any precipitation over the period of at least one week. F-127 plays an important role not only regarding dispersion stability but also regarding the generation efficiency of nanoparticles. It is considered that the surface of nanoparticles generated by laser fragmentation is coated rapidly and tightly with F-127 molecules through hydrophobic interactions between the hydrophobic nanoparticles’ surface and the hydrophobic PPO block of F-127. A nanoemulsion having a core (MPcs or PtOEP nanoparticle)–shell (F-127) structure formed immediately after laser fragmentation of microcrystals, leading to the generation of highly dispersive nanoparticles with high conversion efficiency. We have demonstrated the photosensitized ROS generation of the AlClPc, ZnPc, and PtOEP nanoparticles prepared by PLAL using 0.1 wt % F-127 aqueous solution as a liquid medium and confirmed their photocytotoxicity for PC12 and HeLa cells. The nanoparticles could be useful as a potential photosensitizer for PDT treatments in biomedical applications.

## Experimental

### Materials

Cobalt(II) phthalocyanine (CoPc, 97%), nickel(II) phthalocyanine (NiPc, 85%), platinum octaethylporphyrin (PtOEP, 98%), and Pluronic^®^ F-127 were purchased from Sigma-Aldrich. Chloroaluminum phthalocyanine (AlClPc, 98%), iron(II) phthalocyanine (FePc, 95%), zinc phthalocyanine (ZnPc, >95%), and 1,3-diphenylisobenzofuran (DBPF) were purchased from TCI Chemicals. All materials were used without further purification. Aqueous solution samples were prepared with deionized water.

### Fabrication and characterization of nanoparticles

Different concentrations of MPcs and PtOEP (0.020 wt % and 0.005 wt %) were prepared as aqueous suspensions in F-127 solutions (0, 0.1, and 5 wt %). The suspensions were sonicated for 20 min with an ultrasonic cleaner (ASU CLEANER, ASONE), then 2 mL of a suspension was placed in a plastic cuvette (1 × 1 × 4 cm^3^) and exposed to an unfocused beam of the second harmonic pulses (532 nm wavelength, 6 ns pulse width, 10 Hz repetition rate) from a nanosecond Nd^3+^:YAG laser (Surelite I, Continuum) at a laser fluence of 140 mJ·cm^−2^ per pulse. The suspension was stirred with a magnetic starrer during the laser irradiation. The absorption spectrum of the sample was measured with a modular USB spectrometer (USB2000, Ocean Optics), and the sample was irradiated with a laser until the absorption spectrum stopped changing.

The laser fluence of 140 mJ·cm^−2^ per pulse was chosen after experiments regarding the laser fluence dependence for AlClPc. The absorption spectra of the sample after laser irradiation at various fluences for 30 min were compared (Figure S9, Supporting Information File 1). The absorption of the nanoparticles increased with the fluence above a threshold (30 mJ·cm^−2^), and the value saturated at the fluence of 140 mJ·cm^−2^. Also, a long tail at a longer wavelength of 900 nm was observed in the spectra at low laser fluences, which decreased with increase of the fluence. Based on these results, we considered that 140 mJ·cm^−2^ is the laser fluence that turns the raw microcrystalline powder into small nanoparticles with high efficiency.

The particle size was determined by dynamic light scattering measurements (Zetasizer nanoS, Malvern Instruments). In this analysis, the prepared colloids were diluted more than tenfold with ion-exchanged water.

### Dispersion stability

Dispersion stability of the nanoparticles was evaluated by spectral absorption measurements. 0.2 mL of the prepared colloids was put in 1.8 mL of deionized water, PBS (pH 7, Thermo Fisher scientific), MEM (Sigma-Aldrich, M4655-500ML), or RPMI1640 medium (Sigma-Aldrich, R8758-500ML) media, and then the absorption spectra were measured after different standing times. The dye concentration in the sample was about 0.002 wt %, and the concentration of F-127 was 0.01 wt %.

### Photocytotoxicity

The photocytotoxicity of the nanoparticles was examined according to [3–6]. The procedure in the present work is illustrated in Figure S7, Supporting Information File 1. PC12 cells were maintained in RPMI1640 medium (Sigma-Aldrich, R8758-500ML) with 10% horse serum (Gibco, 26050088), 5% fetal bovine serum (Gibco, 10270106), penicillin (100 U·mL^−1^)/streptomycin (100 μg·mL^−1^) (Gibco, 15140122) at 37 °C in 5% CO_2_. HeLa cells were maintained in MEM medium (Sigma-Aldrich, M4655-500ML) with 10% fetal bovine serum, penicillin (100 U·mL^−1^)/streptomycin (100 μg·mL^−1^) at 37 °C in 5% CO_2_. PC12 and HeLa cells were plated at a density of 20,000 cells per well of a 96 well plate (CORNING, 356461) and a PDL-coated 96 well plate (Thermo Fisher scientific, 167542), respectively, and the cells were grown overnight in 80 μL medium. Then, 20 μL of nanoparticle colloids diluted with PBS (Thermo Fisher Scientific) to various concentrations was added to each well. After 24 h of incubation at 37 °C in 5% CO_2_, the samples were illuminated with broadband (500–800 nm, Figure S6, Supporting Information File 1) visible-light source at 30 mW·cm^−2^ for 10 min. The cell viability after light irradiation was determined using a MTT cell proliferation kit (Roche) as described in [31–32]. 10 μL of the 3-(4,5-dimethylthiazol-2-yl)-2,5-diphenyltetrazolium bromide (MTT) reagent was added to each well. After 4 h of incubation, 100 μL of solubilization solution (10% SDS, 10 mM HCl) was added to each well. After overnight incubation, the absorbance at 562 nm was measured using a plate reader (TECAN Safire^2^). Each sample was assayed in triplicate, and the data were the average of three wells.

The nanoparticle colloids diluted with PBS were sterilized with a filter (Milex-GP SLGP033RS (0.22 µm)), then used. The concentration of nanoparticles in PBS was estimated by optical absorbance measurements, and the value in the culture medium was changed typically from 0 to 30 μM. The concentration of F-127 the medium was less than 0.01 wt %. We have confirmed that F-127 at this concentration is not cytotoxic.

## Supporting Information

File 1 Additional experimental data.

## Data Availability

Data generated and analyzed during this study is available from the corresponding author upon reasonable request.
