# Supplementary material for: Fabrication of metal complex phthalocyanine and porphyrin nanoparticle aqueous colloids by pulsed laser fragmentation in liquid and their potential application to a photosensitizer for photodynamic therapy
Source: Beilstein J Nanotechnol. 2025 Jul 11;16:1088–96. doi: 10.3762/bjnano.16.80 (PMC12256784; doi:10.3762/bjnano.16.80)
Supplement: File 1 — Additional experimental data. [file Beilstein_J_Nanotechnol-16-1088-s001.pdf]

BEILSTEIN JOURNAL OF NANOTECHNOLOGY

Supporting Information

for

Fabrication of metal complex phthalocyanine and porphyrin nanoparticle aqueous colloids by pulsed laser fragmentation in liquid and their potential application to a photosensitizer for photodynamic therapy

Taisei Himeda, Risako Kunitomi, Ryosuke Nabeya, Tamotsu Zako and Tsuyoshi Asahi

Beilstein J. Nanotechnol. **2025,** *16,* 1088–1096. doi:10.3762/bjnano.16.80

Additional experimental data

License and Terms: This is a supporting information file under the terms of the Creative Commons Attribution License (https://creativecommons.org/licenses/by/4.0). Please note that the reuse, redistribution and reproduction in particular requires that the author(s) and source are credited and that individual graphics may be subject to special legal provisions.
The license is subject to the *Beilstein Journal of Nanotechnology* terms and conditions: (https://www.beilstein-journals.org/bjnano/terms)

Absorption spectra of nanoparticle colloids and dispersion stability

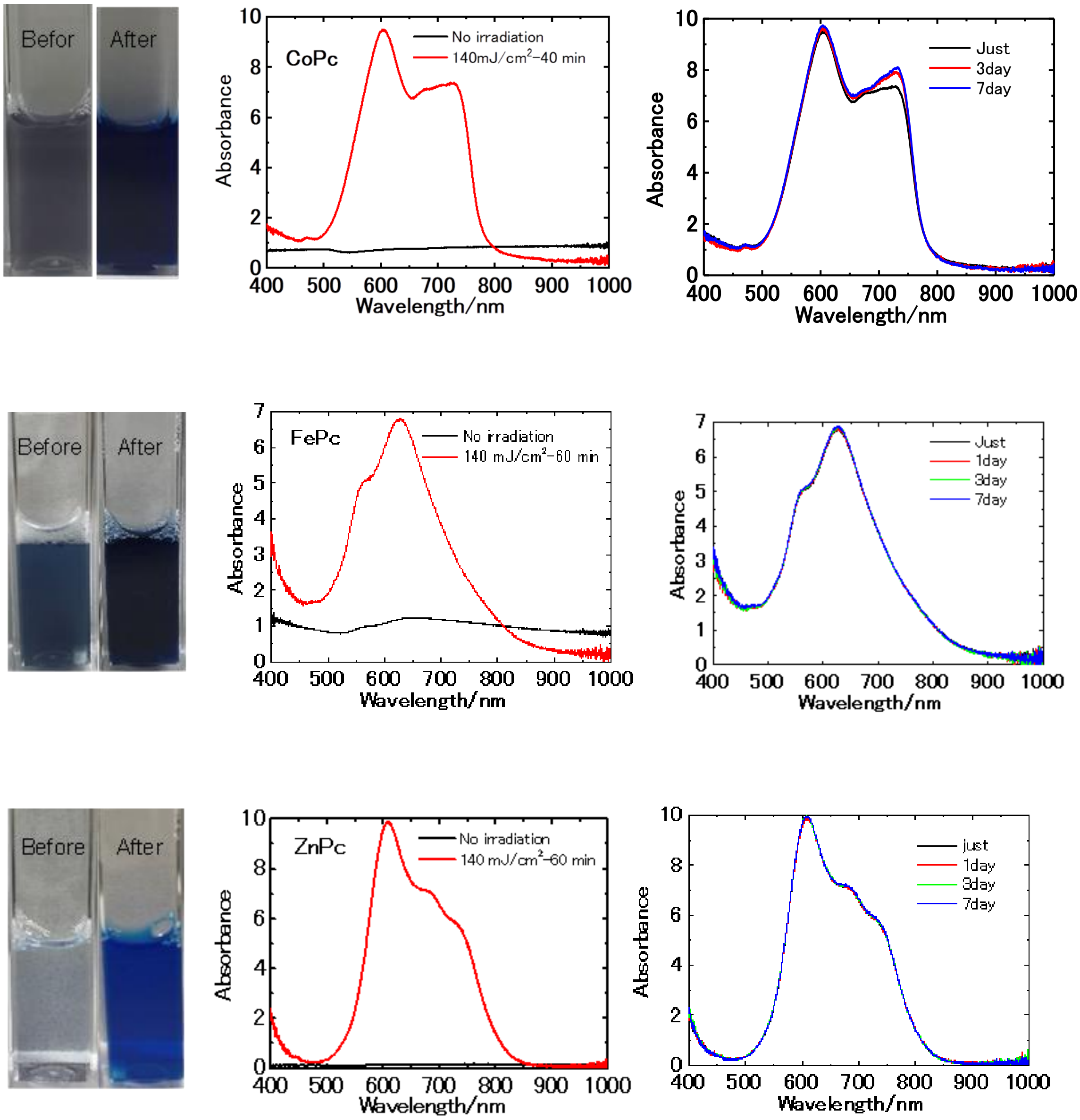

Figure S1: (Left row) Photographs of the mixtures of MPc (0.020 wt %) and F-127 (0.1 wt %) aqueous solutions before and after nanosecond pulsed laser irradiation (532 nm wavelength, 6 ns full-width at half-maximum, 10 Hz repetition rate) at 140 mJ/cm^2 for 30–40 min. (Middle row) Absorption spectra of the mixtures before (black line) and after (red line) laser irradiation. (Right row) Absorption spectra of the prepared nanoparticle colloids at different standing times for a total of one week after laser irradiation.

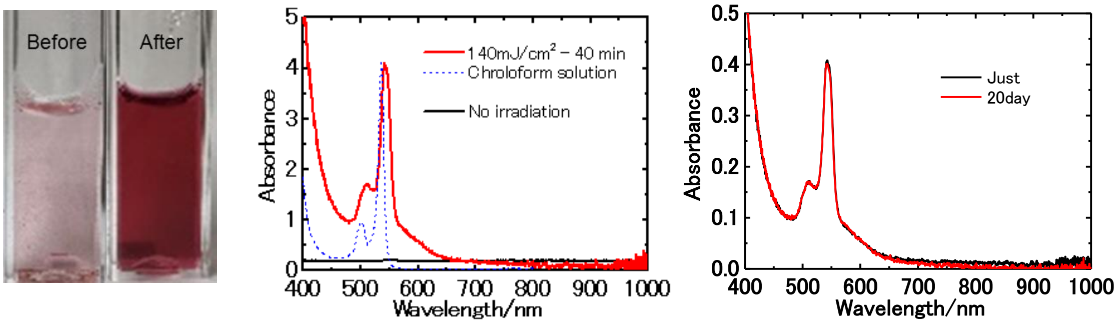

Figure S1 (continued): (Left) Photographs of the mixture of PtOEP (0.020 wt %) and F-127 (0.1 wt %) aqueous solution before and after nanosecond pulsed laser irradiation (532 nm wavelength, 6 ns full-width at half-maximum, 10 Hz repetition rate) at 140 mJ/cm^2 for 30 min. (middle) Absorption spectra of the mixture before laser irradiation. (Right) Absorption spectra of the prepared nanoparticle colloids at 10 min and 20 days after laser irradiation.

Number-weighted particle size distribution of nanoparticles

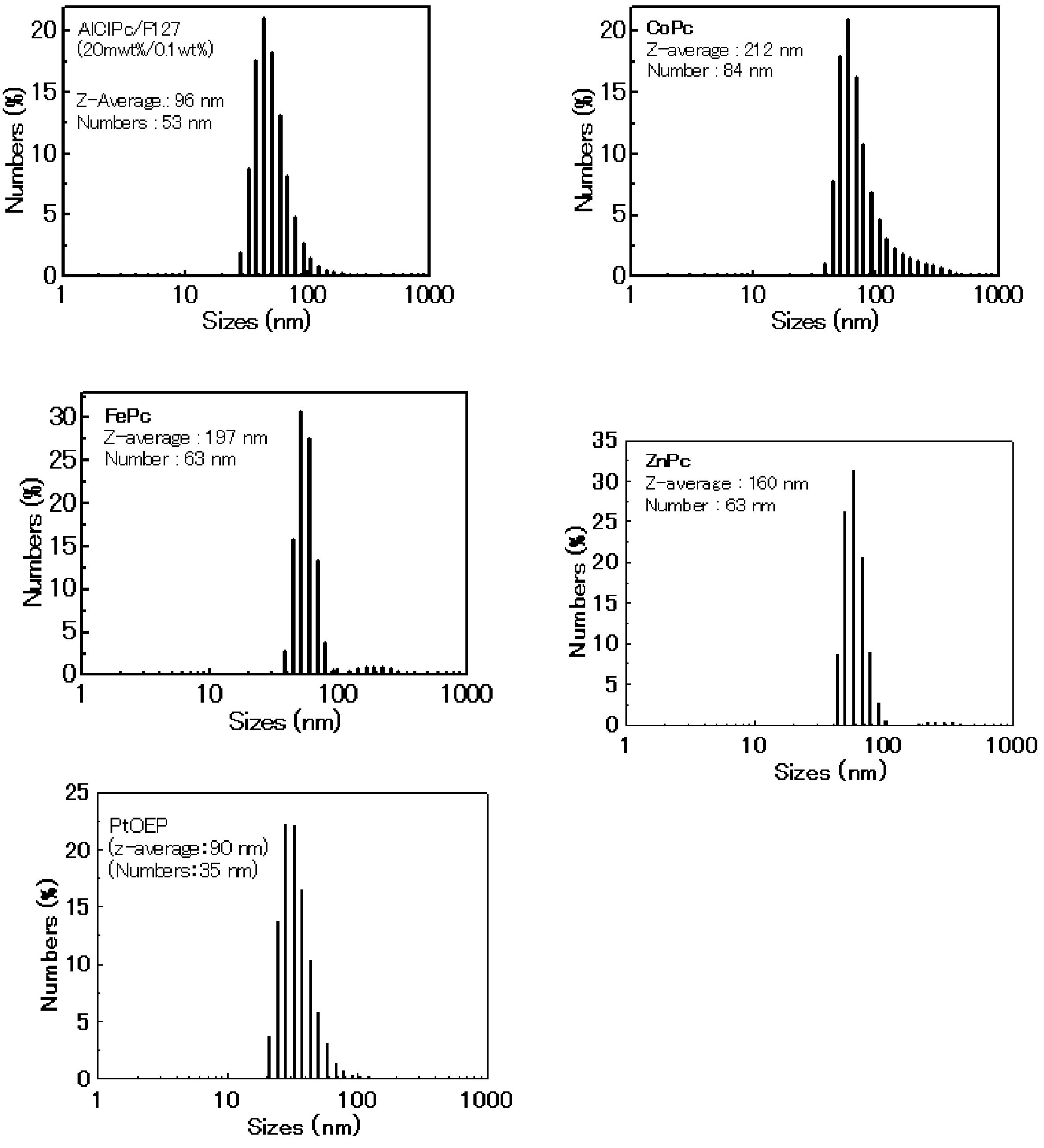

Figure S2: Number-weighted particle size distribution of AlPc, CoPc, FePc, ZnPc, and PtOEP nanoparticles prepared by laser irradiation of their microcrystalline powder (0.020 wt %) suspended in F-127 (0.1 wt %) aqueous solution. The prepared colloids were diluted more than tenfold with deionized water and measured.

Dispersion stability of nanoparticles in PBS and cell culture media

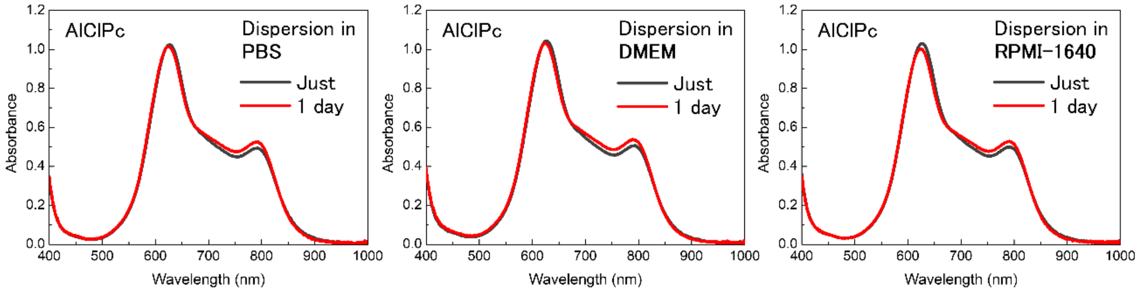

(a)

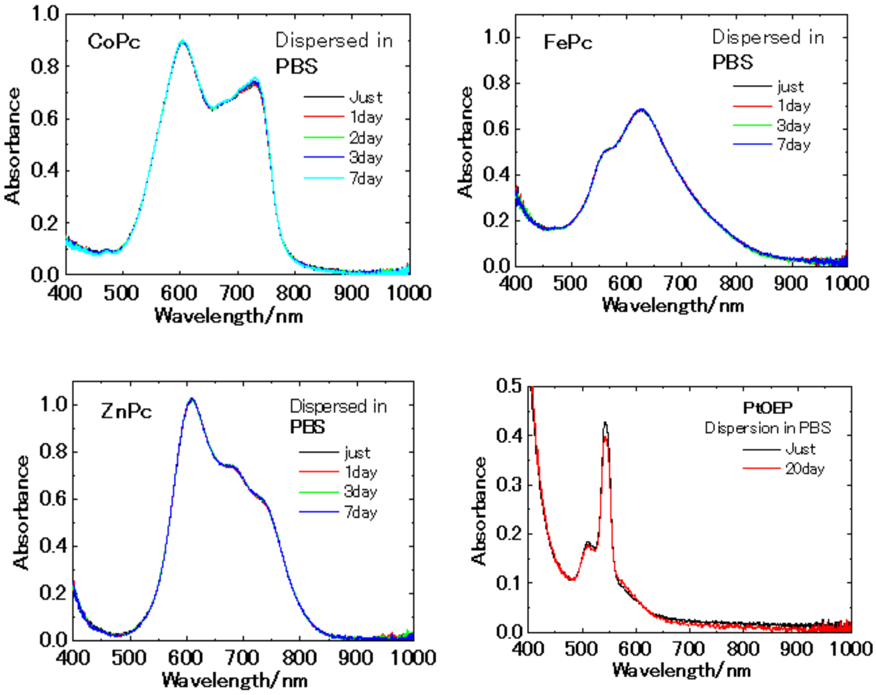

(b)

Figure S3: (a) Absorption spectra of AlClPc nanoparticles in PBS, DMEM, and RPMI1640 at 10 min and one day after mixing 0.2 mL of the fabricated colloid with 1.8 mL of each medium. (b) Absorption spectra of CoPc, FePc, ZnPc, and PtOEP nanoparticles in PBS with different standing times after mixing 0.2 mL of the fabricated colloid with 1.8 mL of PBS. The nanoparticle colloids were fabricated by nanosecond laser irradiation of the mixtures of dye powder (0.020 wt %) and F-127 (0.1 wt %) aqueous solution at a laser fluence of 140 mJ/cm^2.

Effect of F-127 on nanoparticle formation and dispersion stability

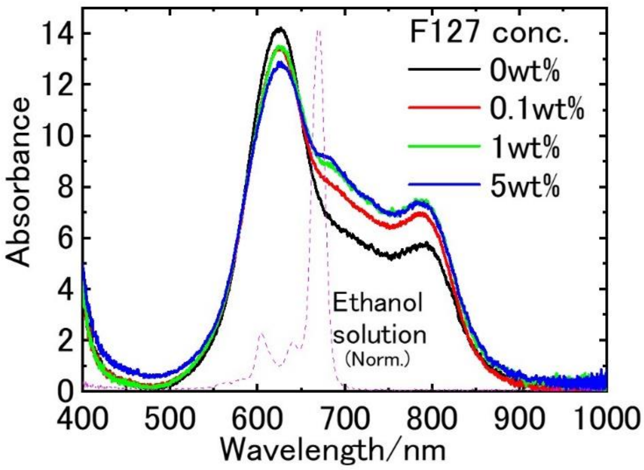

Figure S4: The addition of F-127 does not significantly affect the formation of AlPc nanoparticles since the absorbance of the materials after laser irradiation is similar. The slight difference in the spectral shape depending on the concentration of F-127 is due to changes in the packing of the molecules in the nanoparticles. The nanoparticle colloids were prepared by laser irradiation to the mixture of AlClPc (0.020 wt %) and F-127 (0.1 wt %) aqueous solution at 140 mJ/cm^2 for 30 min.

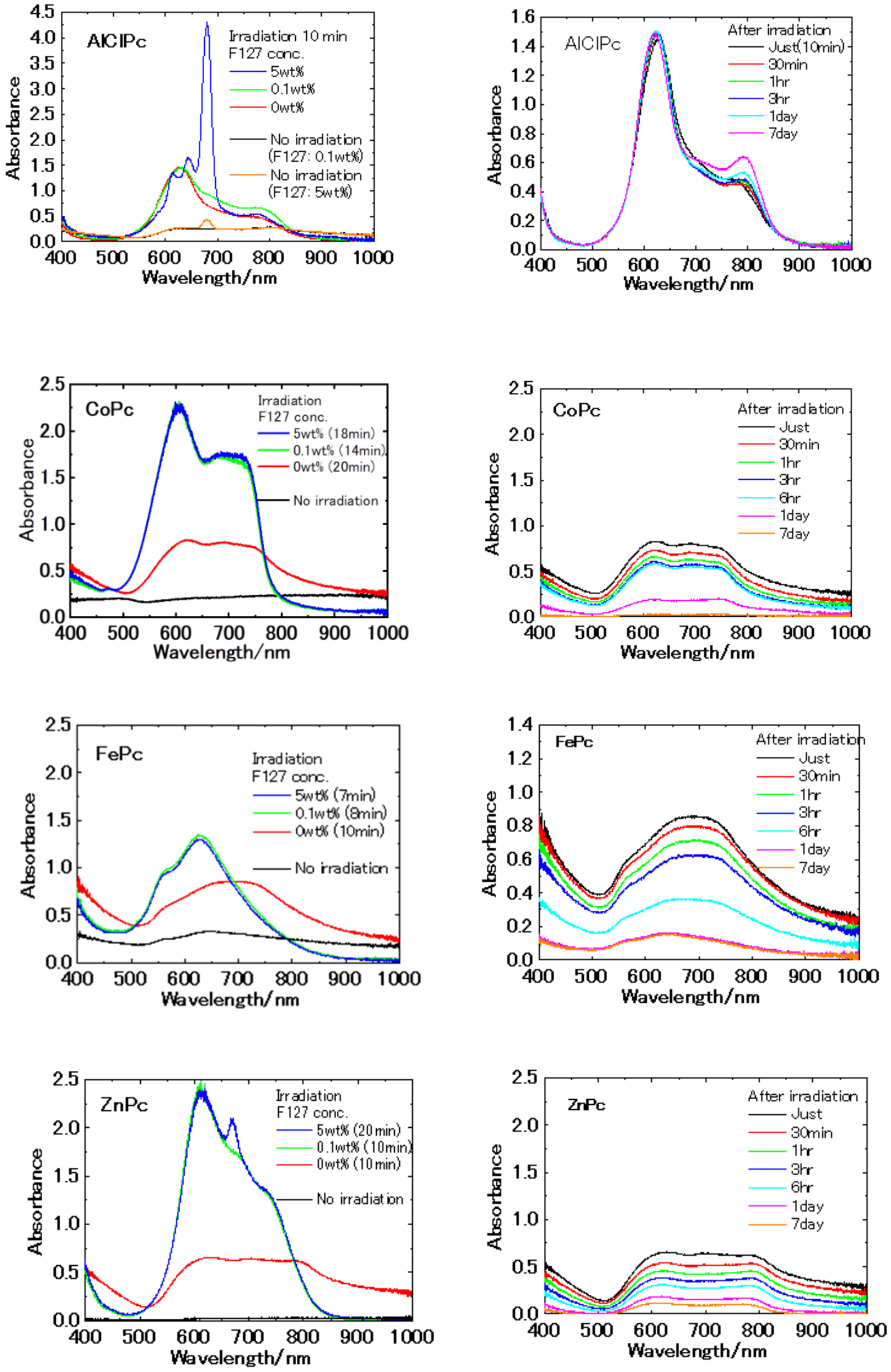

Figure S5: (Left row) Absorption spectra of the MPc (M = AlCl, Fe, Co, Zn) colloids (0.005 wt %) fabricated in F-127 solutions of different concentrations (0, 0.1, 5 wt %) with nanosecond pulsed lasers at 140 mJ/cm^2 for 20 min. (Right row) Absorption spectra of the pure MPcs colloid prepared without F-127 after being left standing for one week.

Photocytotoxicity experiments

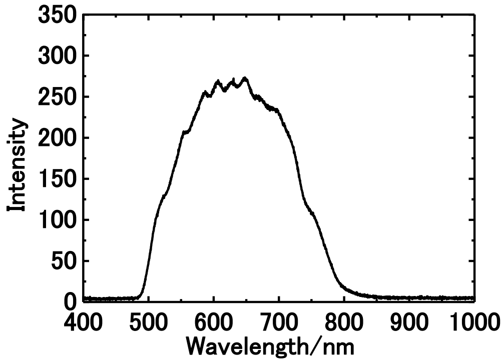

Figure S6: The spectrum of the visible-to-NIR light for photocytotoxicity and photosensitized ROS generation experiments.

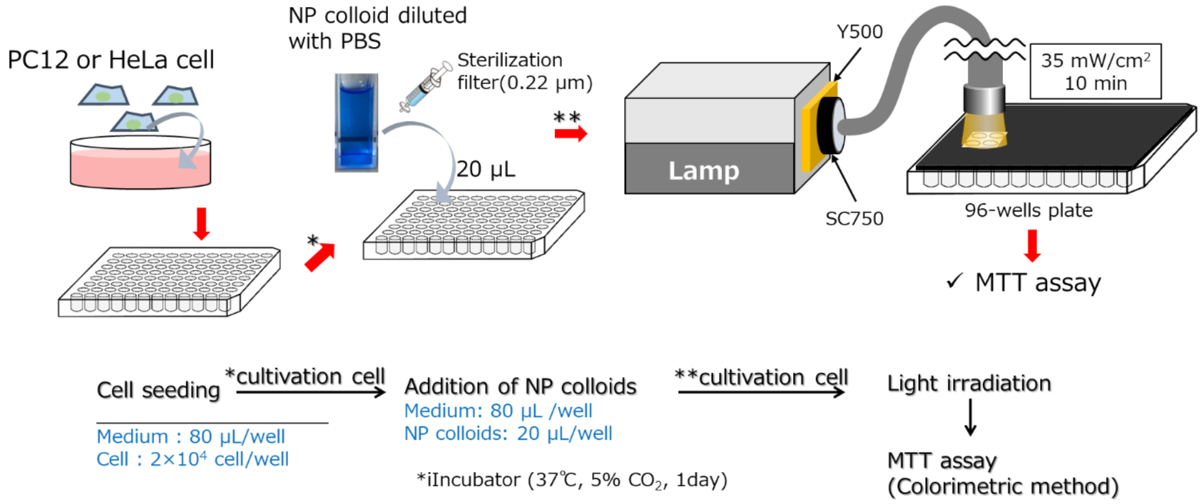

Figure S7: Illustration of photocytotoxicity experimental procedure.

Comparison of the photosensitized ROS generation between PtOEP nanoparticles and the water-soluble metal porphyrin ZnTPPS

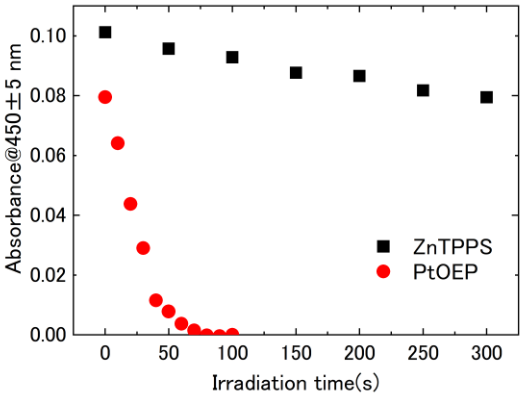

Figure S8: The generation of ROS monitored by absorption spectroscopy as the photolysis of DPBF (1,3-diphenylisobenzofuran). The decay profiles of DPBF absorption at 450 nm for the PtOEP nanoparticle colloid and the ZnTPPS aqueous solution under irradiation of 540 nm light at 5 mW. [PtOEP] = 25 μM, [ZnTPPS] = 20 μM, and [DPBF] = 25 μM. The absorbances of the samples were the same at 540 nm. A rapid decay of DPBE absorption in PtOEP colloid demonstrates clearly that the nanoparticles acted as a photosensitizer with greater efficiency than ZnTPPS.

Laser fluence dependence of nanoparticle generation for AlClPc evaluated by absorption spectral measurements

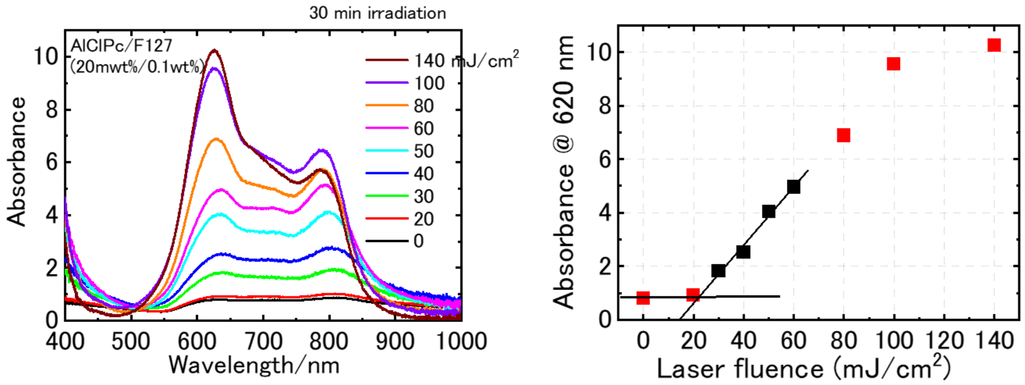

Figure S9: (Right) Absorption spectra of the mixture of AlClPc (0.020 wt %) and F-127 (0.1 wt %) aqueous solution after nanosecond pulsed laser irradiation (532 nm wavelength, 6 ns full-width at half-maximum, 10 Hz repetition rate) at various laser fluences for 30 min. (Left) Laser fluence dependence of absorbance at 620 nm. The absorption increased with the fluence above a threshold (30 mJ/cm^2), and the value saturated at the fluence of 140 mJ/cm^2. A long tail at a longer wavelength of 900 nm was observed in the spectra at low laser fluences, which means the generated nanoparticle size is large. The absorbance due to light scattering of particles decreased with increase of the fluence, and close to zero, which means the nanoparticle size became small, and almost raw microcrystalline powder converted into the small nanoparticles. The shape of the absorption spectrum depended on the laser fluence, with a change in relative intensity at wavelengths of 620 and 800 nm. This is probably due to changes in the crystal structure of the nanoparticles. The absorbance spectrum of the nanoparticle colloid gives the sum of the absorption and scattering spectra of the nanoparticles. The larger the size of the nanoparticles, the greater the scattering contribution, giving a size-dependent absorbance spectrum. This is another reason of the observed fluence-dependent spectra.
